# Remarks on Intra-Pelvic Inflammation in the Chronic Form

**Published:** 1883-10

**Authors:** W. H. Byford

**Affiliations:** Chicago, Professor of Gynæcology, Rush Medical College


### Selections.

---

REMARKS ON INTRA-PELVIC INFLAMMATION IN THE CHRONIC FORM.—By W. H. BYFORD, M.D., of Chicago, Professor of Gynæcology, Rush Medical College. (Presented to the Section on Obstetrics and Diseases of Women of the American Medical Association.)

The terms parametritis and perimetritis are erroneously supposed by many to include the whole subject of intra-pelvic inflammation. These terms are misleading, because as now often used they prosent to the mind the idea that all cases of inflammation not confined to the uterus must belong to one or the other of these.

Actual observation teaches the important fact that perimetritis and parametritis generally exist together, and that they are usually complicated with inflammation of the uterus, and not infrequently the ovaries and fallopian tubes are involved. When we use the terms perimetritis and parametritis, if anything like a definite diagnosis is made we ought to understand that the greater *intensity* of lesion is manifested in one of the tissues, but that inflammation extends to the others also. It is apparent, therefore, that to determine the tissue in which the inflammation is located is often difficult, simply because uncomplicated cases are extremely rare.

The complexity of the lesions of the pelvic organs and tissues might be inferred from the almost absolute unity of the vascular and nervous supply, and the fact that the genito-spinal center is the common controlling influence.

I make these general remarks upon the pathology of intra-pelvic inflammation, as an introduction to what I have to say of the various forms of its manifestation in different organs and tissues within the pelvis.

The more obvious conditions of chronic parametritis are:

First.—Suppuration, or chronic pelvic abscess, located more frequently, but not always, in the broad ligament, the consequence of cellulitis. Chronic purulent accumulations are often found also behind the uterus, and are doubtless the result of local peritonitis.

The chronic pelvic abscess is generally the sequel of acute inflammation, and attains chronicity from the imperfect evacuation of the pus after acute inflammation has terminated in suppuration. The discharge in these cases may be continuous, but the suppurating cavity, in not being completely evacuated, is consequently not obliterated.

The evacuation is deficient sometimes because the outlet is through a tortuous canal; at others because the termination of the canal is in the rectum or bladder. The muscular fibers of the walls of these receptacles, after a certain amount of pressure is taken off by partial evacuation, contract around the opening, and do not yield until the accumulation renews the pressure sufficiently to overcome their resistance. These processes of partial evacuation and accumulation are repeated indefinitely. Again, temporary interruption of the purulent discharge may be caused by the distal extremity of the evacuating tube being higher than some portion of the suppurating cavity.

If the abscess is located in the connective tissue, the elasticity of that tissue will very materially diminish the size of the suppurating cavity each time the evacuation takes place, and eventually may entirely obliterate it. It does not always do so, however. The sufferings experienced by the patient in these cases consists in pyæmic and septic symptoms, resulting from the resorption of the pus and debris of decomposing material contained in the cavity. While the vital energies of many patients will sustain them until the process of evacuation is completed, others will die from exhaustion.

The remedy in such cases is found in surgery, and consists in making a more direct outlet through the vagina, large enough to at once completely evacuate the pus and enable the surgeon to cleanse and disinfect the cavity. Where the evacuating canal is tortuous or too small it may sometimes be dilated with instruments until the cavity can be evacuated and washed out. Where the pus is accumulated in a sac formed in the peritoneal cul-de-sac behind the uterus, the difficulty will not be so easily overcome by enlarging the opening, as the pyogenic cavity is not surrounded by elastic tissue, as in the broad ligament. It will, as a consequence of this latter fact, require a longer time to fill up by granulation. In such cases we may hasten granulation by stimulating the cavity with injections of a weak solution of permanganate of potash, swabbing it out with iodoform or some other stimulating remedy. A cavity situated in this position will bear such stimulation better than one situated in the broad ligament. In cavities thus located we will often find decomposing coagula the remains of a hematocele, the most common origin of abscesses in this part of the pelvis.

The suppurating variety of intra-pelvic inflammation may, however, be primarily chronic, and never rise above that grade.

In such cases the inflammation persists for several years. A considerable quantity of pus is formed without hectic or other symptoms of absorption, save a general feeble state of health. In these indolent abscesses the pus is formed and remains enclosed in the cavity for a very long time, with, in fact, very little tendency to evacuation. I saw one case of this kind in which I know from observation that pus was retained for three years. Upon first seeing the patient I was so well assured of the presence of pus in the right broad ligament that I proposed to puncture it. The patient, however, declined, and, residing in a distant city, went home. Three years afterward I made an opening in it, and removed a quantity of dirty putrid sero-pus. The patient was then in an advanced stage of phthisis, and died in a few months.

Another case I saw with Dr. T. D. Fitch. The patient assured us that the tumor had been discovered seven years before by her medical attendant, who told her she had cellulitis of the broad ligament, which had already suppurated. There were eight ounces of the same thin pus, as in the former case.

A cavity that has contained pus for so lomg a time has ceased to be pyogenic; its walls are thin and yielding over a large surface, and when evacuated has no tendency to fill up again. These cases are sometimes connected with the tubercular diathesis.

Second.—Chronic parametritis is often met with when there is a decided tumefaction—in one of the broad ligaments, more frequently than otherwise—the remains of an acute attack, and, when not noticed or attended to properly, is a latent focus of phlogosis that may easily be elevated into the acute grade. We frequently meet cases of this kind among poor patients, who are obliged to exert themselves more than is compatible with the process of resolution. The consequences are that the woman has repeated returns of acute attacks until suppuration takes place. Sometimes in these or even more favored patients injudicious thoroughness and earnestness in an examination to ascertain the exact condition of the patient develops an acute inflammation that inflicts fearful suffering, and perhaps cripples the patient for life. Or, again, without sufficiently careful examination to ascertain the existence of this nodule of inflammation, irritating applications are made to the uterus, or a pessary is introduced that presses upon the locality of the effusion, or the womb is lifted high enough to make traction upon the diseased ligament, to give origin to acute inflammation. When the tumor is small, or in some remote part of the ligament, these mistakes are very liable to occur. There is no doubt also that in numerous instances these effusions are primarily the results of chronic inflammation, and have for their origin causes operating less violently. After the absorption more or less completely of the exudate in such cases, the broad ligament may be left thickened, rigid, shortened and irritable for a long time, and become the nidus for an acute attack.

Careful manipulation will enable us to discover any of these conditions without doing damage. They should always be the object of treatment, and removed before treatment for chronic metritis or replacement of the uterus is safe or justifiable.

The proper local treatment is hot-water injections and counter-irritation.

The general management is of the first importance, and consists of alteratives, tonics, feeding and rest. We should expect and require a long course of treatment for the cure of cases of this kind.

Third.—Chronic local *peritonitis* may exist (either as a *separate*

*disease* or a complication of cellulitis, ovaritis, and salpingitis) in the retro-uterine *cul-de-sac*, and over the broad ligaments retaining a displaced uterus in a fixed, retroversed or retroflexed position. The fixed position may be maintained by a somewhat solid cohesion of the opposed surfaces of the peritoneal covering of the uterus and the posterior lining of the Douglass pouch, or by the extension of bands of false membrane that permits of limited motion. When associated with general chronic pelvic inflammation it is but one item, is obvious and easily detected; but when local peritonitis is the only inflammatory condition it may require much care to diagnose it. When I find the uterus turned backward and resisting reasonable force, I suspect this form of chronic inflammation, and if manipulation for the purposes of restoration gives the patient much pain, I am confirmed in such suspicion. Enlargement of the uterus makes that organ, to a certain extent, difficult of replacement, and attempts to do so will generally cause pain; but even in such a case the presumption is that there is contiguous inflammation, and all the cautions to avoid aggravating that condition should be observed. An examination per rectum will give us valuable information as to the presence of retroversion and local peritonitis. By passing the finger through the rectum up the posterior wall of the uterus we will ascertain the condition of the peritonæum as to sensitiveness. With the evidence we may thus gain, and by gentle attempts to move or replace the uterus, we can make a pretty definite diagnosis. This inflammation may also exist in the cul-de-sac without the malposition of the uterus; in this case the tenderness behind that organ will be sufficiently diagnostic.

Sometimes we find local inflammation limited to the vesico-uterine reflexion of the peritonæum, which may interfere with reposition from anteflexion or anteversion of the uterus. In such cases we will find any effort to move the uterus attended with pain, and there will generally also be vesical irritation of an absolute character.

The discovery of peritoneal inflammation in however slight a degree should be a matter of caution to us against free manipulation for any purpose, and when complicating displacements, subinvolution or chronic inflammation of the uterus should be the main subject of our attention until entirely removed. I am quite sure that the lack of sufficient care in this particular has been the cause of much needless suffering.

This remark, I think, is especially applicable to efforts at replacements of the uterus when that organ is retroverted or anteverted. I would therefore emphasize the direction, *not to try to replace* the uterus when such attempts give the patient decided pain. Counter-irritation, hip baths, and large tepid water injections are the main items of local treatment, while the general consists of alteratives, rest and tonics. The latter is of special importance. In many cases nourishment will be more valuable than medicine, as a large number of these patients are profoundly anæmic.

Fourth.—Another condition which accompanies a great number of cases, is inflammation of the ovaries and fallopian tubes. The inflammation of the ovary and tube is not often completely isolated, but is a complication of a more diffuse lesion of the broad ligament, including most of its structures. When ovaritis and salpingitis, one or both, are the only manifestation of existing inflammation, and stand apparently alone, there will be a history of preceding inflammation of the surrounding tissues.

The most important, as well as the most frequent of lesions, are indurated deposits of lymph, rendering the ligament rigid and deformed, and false membranes or trabeculæ that fix the ovary especially, and sometimes surround it in such a way as to constrict the nervous and vascular apparatus.

The ovary thus embraced in semi-organized exudation, if its structure is not completely destroyed, is so mutilated that its functions are greatly deranged, and performed with such difficulty as to cause intense local and general suffering.

According to Mr. Lawson Tait, the fallopian tubes are often the seat of chronic suppurative inflammation, which accompanies and outlasts the chronic inflammation of the ovaries. Mr. Tait regards the disease of the fallopian tubes as a more important factor in the reflex and local sufferings, as well as menstrual derangements, than that of the ovaries. While the position that the morbid condition of the fallopian tubes produces greater menstrual disorder than disease of the ovaries is a subject of controversy, it must be admitted that diseased tubes have a share in causing some of them at least, and I think Mr. Tait is right in concluding that in cases of oöphorectomy it is quite as necessary for the relief of the patient to remove a diseased fallopian tube as an unsound ovary. This is not, however, admitting that the tubes in a healthy condition have any direct effect in exciting or in any way regulating the menstrual flow. It has long been a demonstrated fact that inflammation in the broad ligament, and other portions of the pelvic tissues, gives rise to pain during menstruation and causes general hystero-neuroses.

The symptoms of inflammation situated in the ovaries and fallopian tubes are, to a great degree, like those caused by disease of the uterus and perimetric tissues. If there are any symptoms more than ordinarily distinctive of chronic ovaritis it is the suffering during the menstrual period, or the diminuation or complete suppression of the menstrual flow.

Sometimes, indeed, connected with ovarian inflammation, there is complete amenorrhœa without any suffering at the periods, or any great amount of derangement of the general health. Gynæcologists not unfrequently meet with cases like the following, viz.:

A young lady (27 years of age) at the age of 20 had a severe attack of pelvic inflammation that continued about three months, and, after its subsidence, for several months longer she was the subject of moderate pelvic symptoms.

When entire immunity had come about she observed that her menstrual flow was very much reduced in quantity.

For three years she enjoyed a fair degree of health and was able to exercise her vocation as a teacher with her usual comfort. At the end of that time, from exposure during severe exercise, she was again attacked with symptoms of acute pelvic inflammation, in all respects, so far as she could remember, similar to the first. From the inception of the last attack to the present time the menses have been entirely suspended, and yet she is now in the enjoyment of robust health.

From the history of this case I think we can fairly infer that both ovaries were the subject of inflammation of such a character and degree as to damage their structure sufficiently to render them incapable of performing their functions.

More frequently, however, the stroma is not so greatly changed ; then the functions of the ovaries are performed with great difficulty, and attended with local pains and extensive and intense reflex suffering. To the symptoms of the latter condition the term ovarian dysmenorrhœa is correctly applied.

Rest, local depletion—in the earlier stages—and alteratives are the proper treatment. As the symptoms become chronic we may often derive much permanent good from the effects of one or more setons over the seat of the disease. In some of them the disease is so obstinate and the suffering so great as to justify the removal of the ovaries and fallopian tubes.

Fifth.—We may have cases of slight diffuse, or circumscribed phlogosis or hyperæsthetic hyperæmia, in which no exudation can be detected, and probably there are no palpable anatomical changes. In this form the nerves and blood-vessels are highly excitable because already under the influence of morbific agents that have been acting a long time upon them, but with a degree of intensity short of that condition called an exciting cause. They are in a state of predisposition.

Whether we are justified in speaking of this state of things as inflammation or not, it is quite certainly a departure from a sound condition, in a direction leading to that process. This is probably what authors mean by the term dormant or latent inflammation. It is an actual morbid condition, possessing the two elements, hyperæsthesia and hyperæmia, from which an exciting cause gives rise to the acute form of inflammatory action.

While the inexperienced may awaken acute suffering by injudicious manipulation or the employment of too strong or improper measures of treatment in some of the other forms of chronic inflammation to which I have alluded; it is this occult variety of disease that, figuratively speaking, betrays the most experienced, skillful, and cautious practitioners into methods of diagnosis and treatment that lead to attacks of acute inflammation, explosive in their suddenness and violence. There is no doubt in my mind that, in its subsidence, acute inflammation sometimes leaves behind it this smoldering susceptibility which lingers for months and even years as a menace to the unwary operator. Without the presence of this relict, or harbinger of acute inflammation, it would have been impossible for our accomplished countryman, Dr. George G. Engelmann, to collect so many terribly interesting cases as he reported to the Missouri Medical Society in a paper read before that body, and published in the September (1880) number of the *American Practitioner*. Many of these cases occurred in the hands of some of the most skillful gynæcologists. We can not, therefore, say that they were the result of recklessness or ignorance.

Can we diagnose cases occurring under this division of the subject with sufficient accuracy to enable us to benefit by the knowledge?

Perhaps not always; but in most instances the careful practitioner will have his suspicions aroused by the history and the objective evidence generally obtainable. Judging from my own observation, I should say that the more dangerous cases were those in which this susceptibility was the result of previous attacks of acute inflammation. The history usually is one of inflammation of the pelvis and lower abdominal organs in months or years gone by. A disease, the nature of which may not have been well understood and treated, and vaguely termed inflammation of the bowels, typhoid or malarial fever.

These by-gone attacks sometimes present so few marked symptoms that their nature can not be definitely deduced from the history of them, especially when given by an ignorant patient. Most of the cases of the severe and sudden attacks, within my observation, have taken place where, it was fair to infer, this lingering susceptibility was the consequence of foregone acute attacks instead of a primary condition.

In such a morbid state, what would seem trivial exciting causes may produce terrible symptoms and disastrous consequences under even cautious management.

The untoward inflammation arising out of this susceptibility is also often the result of the too exclusive mechanical ideas entertained with reference to the management of affections of the uterus and other pelvic organs. Most of the pelvic organs lie within reach of the fingers, and instruments devised for the diagnosis and treatment of their diseases. We may thus be led to regard them as the proper subject for free manipulation, without regard to the fact that they are endowed with vital qualities. We may not govern ourselves in our examinations sufficiently by the complaints of the patient, so much as the desire of finding every possible deviation from the natural position, size and consistence of every organ in the pelvic cavity.

Hence, thoroughness of examination in a mechanical sense is not an uncommon source of danger. In our treatment, the same preponderance of mechanical ideas leads to much mischief. A displaced uterus must be rectified by mechanical means alone, often without sufficient regard to other conditions. I have more than once seen cases where the uterus was fixed by effusion from inflammation, treated by forcible attempts at reposition.

Such attempts, I know, are recommended by men who ought to know better.

As a common practice, it would cause extensive suffering, and fail to be attended by any compensating benefits. Very few intelligent practitioners are so reckless as to disregard the actual existence of inflammation under such circumstances. But many times when the inflammation is so slight as not to give rise to noticeable effusion, and yet be attended with obvious tenderness, mechanical support is resorted to, and the patient exhorted to bear some pain for the good it will bring her to have the uterus kept in position. Such treatment is usually followed by bad results.

Now, to avoid mischief from the use of mechanical support in uterine displacements, the practitioner should consider all cases in which even slight perimetric inflammation exists as unsuitable for the pessary. And when the sensitiveness characteristic of inflammation exists to a moderate degree, the uterus should not be reposited. Other treatment should be instituted and persevered in until that sensitiveness is removed, before reposition and mechanical support are resorted to. The partisan advocates of the pessary may think this is unnecessary precaution ; to which I would reply, that while skillful gynæcologists may sometimes disregard this cautious view of the subject, and obtain tolerance of the pessary, even the best of them will sometimes do mischief enough to more than counteract the good they can thereby accomplish.

Our mechanical views as to the treatment of stenosis and flexions of the uterus are apt to betray us into more dangers than those above mentioned. When this slight predisponent condition is present the use of the sponge, seatangle or other dilating tent is frequently followed by great danger ; and it should be remembered that the use of the tent causes this predisposition to inflammation, so that in the consecutive application of tents in any case the second and third become instruments of extreme danger. It is true that in some cases the patient escapes when extra precautions and skill are used, but it is also true that in other cases, notwithstanding all just precautions, terrible results follow.

Now, in all this, I desire to be understood as incalculating the idea that the most accomplished—not alone the ignorant practitioner—may occasionally produce the damaging conditions so appalingly delineated in the paper by Prof. Engelmann above alluded to. Similar remarks are applicable to the use of the stem pessary, either with or without the incisions of the cervix uteri. In estimating the value of mechanical treatment of the uterus we must take into consideration these *exceptional* cases. No man is skilled enough to ignore the fact that he cannot resort to these measures without great hazard to his patient. His practice must be governed by the recognition of the *possible* consequences that may follow.

If not warned by his own observation, he should be forewarned by the researches and observations of others. I have been cognizant of numerous instances of disaster in all of the mechanical methods I have mentioned, and many deaths have resulted from the employment of some of them. These untoward cases are usually not published. They ought to be published, however, as danger-signals to warn the unwary of the hazards that beset their paths.

As the main object I had in view in writing this paper was to caution my associates against the dangers of converting a chronic pelvic inflammation into a disastrous acute form, I desire to append a summary of suggestions and inferences drawn from it.

1. The sometimes terrible effects of examinations or operations in the pelvis do not often, if ever, take place when there is not a perceptible predisposing inflammation.

2. The inflammation may be so slight as to be easily overlooked.

3. It may be an original condition; the sequence of an acute attack long gone by, or it may be the product of some immediately previous examination or operation, the effects of which have not subsided.

4. To avoid the dangers of acute inflammation we should, in making a first examination for pelvic disease, conduct it in such a way as not to give the patient much pain, and when she complains of much suffering desist at the sacrifice of completeness of diagnosis.

5. Complaints of much tenderness to the touch, or the use of instruments; especially in parous women, is sufficiently diagnostic of inflammation upon which to base treatment for that condition.

6. If, with such tenderness, a thorough examination or an operation is imperative, it should be done under profound anæsthesia. There is no question, in my mind, that much less danger of ill effects is incurred in making examinations or operations on susceptible subjects, under the free use of anæsthetics.

7. Examinations or operations should not be repeated until the effects of the first have entirely passed off.

8. As chronic parametritis is a frequent complication of most of the morbid conditions of the uterus, it should be always suspected and its diagnosis be carefully considered in all cases of metritis.

9. When chronic parametritis is present, it should be the chief, if not the exclusive object of treatment until removed.

10. It is not safe to use the sound, sponge-treatment, or intrauterine stem when there is perimetric inflammation.

11. It is especially dangerous to replace a displaced uterus, when it is bound down by inflammatory adhesions, by any means which will overcome its fixedness by force.

12. The use of pessaries, or supports of any kind which find their lodgement in the pelvis, is generally followed by disastrous consequences when there is even slight primitive inflammation.

13. All local treatment of the uterus must be conducted with the greatest care in all cases where this complication is present. —*Journal of the American Medical Association.*

